# Individual Revision Knee Arthroplasty Is a Safe Limb Salvage Procedure

**DOI:** 10.3390/jpm11060572

**Published:** 2021-06-18

**Authors:** Peter Savov, Lars-Rene Tuecking, Henning Windhagen, Max Ettinger

**Affiliations:** Department of Orthopedic Surgery, Hannover Medical School, Anna-von-Borries-Strasse 1–7, 30625 Hanover, Germany; lars-rene.tuecking@diakovere.de (L.-R.T.); henning.windhagen@diakovere.de (H.W.); max.ettinger@diakovere.de (M.E.)

**Keywords:** custom-made, rTKA, 3D-printed, individual, limb-salvage, cone

## Abstract

Introduction: Revision total knee arthroplasty after multiple pre-surgeries is challenging. Due to severe bone defects, standard implants for metaphyseal and diaphyseal anchoring may no longer be suitable. The primary aim of this case series is to evaluate the early complication rate for individual knee implants with custom-made cones and stems after two-stage revision with severe bone defects. Methods: Ten patients who were treated with custom-made 3D-printed knee revision implants were included. Inclusion criteria were a two-stage revision due to late-onset or chronic periprosthetic joint infection as well as aseptic loosening. All severe bone defects were AORI type III. All procedure-related complications were evaluated. Postoperative range of motion after one year was measured. The time between the two surgeries was evaluated. Results: The mean follow-up was 21 months (range: 12–40). The mean time between the two-stage surgeries was 71.6 days. No fractures were observed intra- and postoperatively. Two patients were revised without changing metal components due to persistent hematoma (three weeks post-surgery) and persistent PJI (three months post-surgery). The mean passive postoperative range of motion was 92° (range: 80–110°). Conclusions: Individual custom-made implants for rTKA provide a safe procedure for patients with huge bone defects after several pre-surgeries. If standard knee systems with standard cones or sleeves are not suitable anymore, custom-made treatment offers the patient the last option for limb preservation. However, this is associated with increased costs.

## 1. Introduction

Due to rising numbers of total knee arthroplasties (TKA) for the treatment of osteoarthritis (OA) of the knee joint, the numbers for revision total knee arthroplasty (rTKA) are analogously rising. In Germany, 14,462 revision surgeries were performed in 2019. At least one metal component was changed in 57.8% of the cases. This is an increase of 3% compared to 2018 [1]. A projection for the United States of America indicates an increase of 601% of rTKA between 2005 and 2030 [2]. In 31.2% of all revisions being performed in Germany in 2019, a condylar constrained knee (CCK) or rotating-hinge implant was needed to stabilize the knee. The correct diagnosis and the operative plan require a high amount of surgical experience. Likewise, the infrastructure is essential being able to address the reasons for the revision indication [3].

In the past, implant failure and polyethylene wear were the main reasons for rTKA. Currently, aseptic loosening, infections, and instability problems are the primary cause of revisions [1,4]. The primary aim of rTKA is restoring the natural joint line in the frontal and sagittal plane with sufficient anchoring. An elevation of the joint line of 4 mm already reduces significantly the maximal flexion [5,6,7]. Further, a loss of posterior condylar offset (PCO) is also associated with a reduction of the postoperative flexion of the knee joint [8,9].

Increasing numbers of rTKAs in younger patients due to the high amount of primary TKA in patients under the age of 65 leads to additional issues. In the case of aseptic loosening, periprosthetic osteolysis (PROL) becomes a significant problem in the revision procedure. Increased accumulation of osteoclasts at the bone-implant interface, impaired osteoblast function, mechanical stresses, and increased production of synovial fluid lead to bone resorption and subsequent loosening of the implant. One of the main causes for PROL is the activity level of the patient [10].

In case of periprosthetic joint infection (PJI), one- or two-stage surgical revision with an exchange of the implant is needed. In addition to antibiotic therapy, a radical debridement of the situs is essential to ensure infection eradication. However, the preservation of good bone stock is the basis for a successful reimplantation of a new implant [11]. For fixation, two different zones should be used. The articular surface is in most cases insufficient for fixation. To provide good stability, the fixation is based on both the metaphysis and diaphysis. Cemented or cementless stems are used for the fixation in the diaphysis [11,12]. In recent years, sleeves and cones were established for augmentation of metaphysical defects and represent the new gold standard. With those modern techniques, AORI-type-IIb and III defects can be addressed very well [11,13]. In addition, metal augments and bone-impaction grafting are used to restore the native joint line [14].

However, standard instrumentation and implants have limitations when huge bone defects occur. Traditionally, megaprosthesis with a proximal tibial replacement, arthrodesis, or amputation were performed in those cases. With modern titanium alloy 3D printing technologies, CT-based individual implants could be used for major bony defects [15]. Both individual cones and stems can be manufactured. Those individual implants provide a homogeneously distributed bone stress [16]. The CT scan can be performed preoperatively before a one-stage revision or in the interval of a two-stage revision procedure. Thus, megaprostheses with the loss of the tuberosity tibiae with a high early and long-term revision rate can be avoided for those previously difficult to treat cases. For those megaprostheses, a complication rate of 52% is reported after a midterm follow-up [17].

Due to the lack of evidence, a case series was performed to analyze the complication rate for individual knee implants with custom-made cones and stems after two-stage revision with severe bone defects after aseptic loosening and PJI in a single center. The primary hypothesis is that the treatment with individual custom-made knee implants is a safe procedure and provides low complication rates.

## 2. Methods

### 2.1. Patients

**Figure 1:**
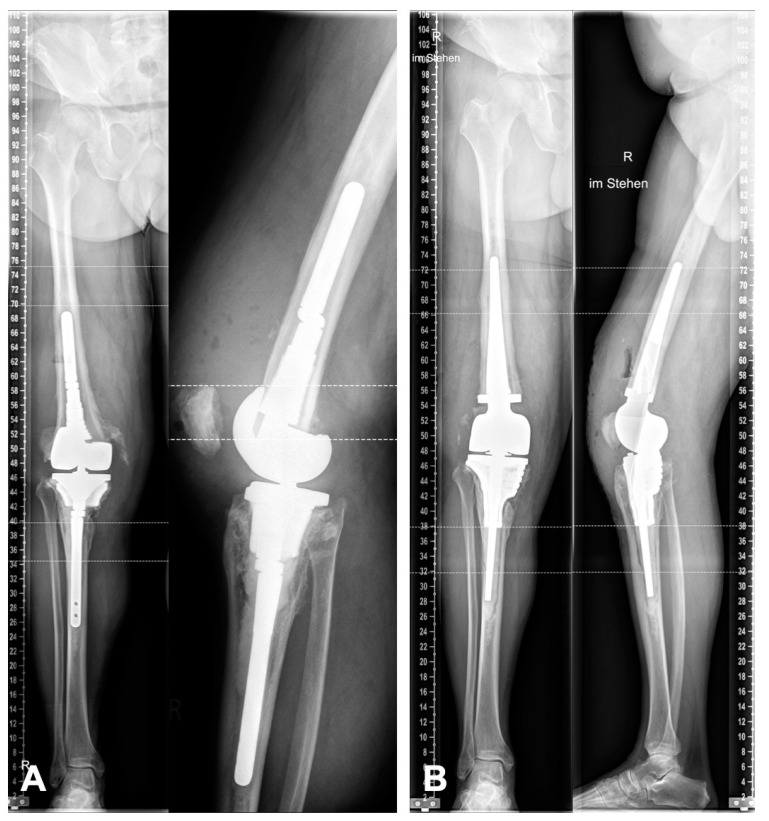
(**A**) Preoperative X-rays before explantation of the septic prosthesis. (**B**) Postoperative X-rays after reimplantation. The joint line and limb alignment are fully reconstructed. (R = right).

Ten patients who were treated with individual custom-made 3D-printed knee revision implants were included. The basis of the knee system was the Link^®^ Endo-Modell rotating-hinge system (RTH) and Megasystem-C distal femoral replacement (DFR) (Waldemar Link GmbH & Co. KG, Hamburg, Germany). All patients were treated between March 2019 and May 2020. The minimum follow-up was 12 months. The mean patient age at the time of surgery was 68.4 years (range 59–79). Inclusion criteria were a two-stage revision due to late-onset or chronic PJI as well as severe bone defect after aseptic loosening. All bone defects were AORI type III and could not be treated with a standard cone or stem (Figure 1A). All patients were treated with an antibiotic static cement spacer in the two-stage revision interval. No aspirations were routinely performed before the second-stage revision. There were no exclusion criteria. All surgeries were performed by a single senior surgeon. Approval for this retrospective case series was given by the institution’s review board (8439_BO_K_2019). Informed consent was obtained from all subjects involved in the study.

### 2.2. Parameters

The indications and characteristics of each implant are displayed in Table 1. The previous surgeries of the patients and all implant costs are displayed in Table 2. The time between the two surgeries was evaluated. All patients received pre- and postoperative standard long leg and true lateral radiographs. The hip–knee–ankle angle (HKA) was analyzed pre- and postoperatively. All procedure-related complications were evaluated including early revision due to seroma, hematoma, or wound healing disorder, intra- and postoperative fractures, early-onset (<4 weeks), and late-onset (>4 weeks) infections. Postoperative range of motion after one year was measured. The pre- and postoperative VAS scale was raised.

### 2.3. Manufacturing

The planning and manufacturing of each implant is based on a CT scan. Close cooperation with the respective engineer of the manufacturer is necessary. Nonmetallic static spacers are recommended for use to increase the quality of the scan with fewer image artifacts. One-stage revisions are possible as well. In those cases, a metal artifact reduction sequence (MARS) should be used.

For severe metaphyseal tibial and femoral defects, hybrid cones are individually manufactured from titanium alloy (TiAl6V4). For the 3D printing process, the electronic beam melting (EBM) or selective laser melting (SLM) technique are used. This is followed by a hot isostatic pressing (HIP) to close unwanted cavities. To achieve improved osteointegration, the cone is coated with calcium phosphate. The surface is highly porous with a structural depth of 2 mm and a pore size of 610–820 µm.

To provide additional stability, individual stems for the femur or tibia with an oval shape can be used. Thus, an almost form-fit anchoring is possible over the entire length of the stem with an additional degree of rotational stability. These stems are usually provided with a taper that allows coupling with a component of a standard implant. An individual printed or standard collar with calcium phosphate coating can be used for the femur implant. Each custom-made stem is made by a CNC machine and comes with an individual rasp. Both cemented and cementless fixation is possible. Further, the implants can be coated with silver by the manufacturer.

### 2.4. Planning and Surgical Technique

**Figure 2:**
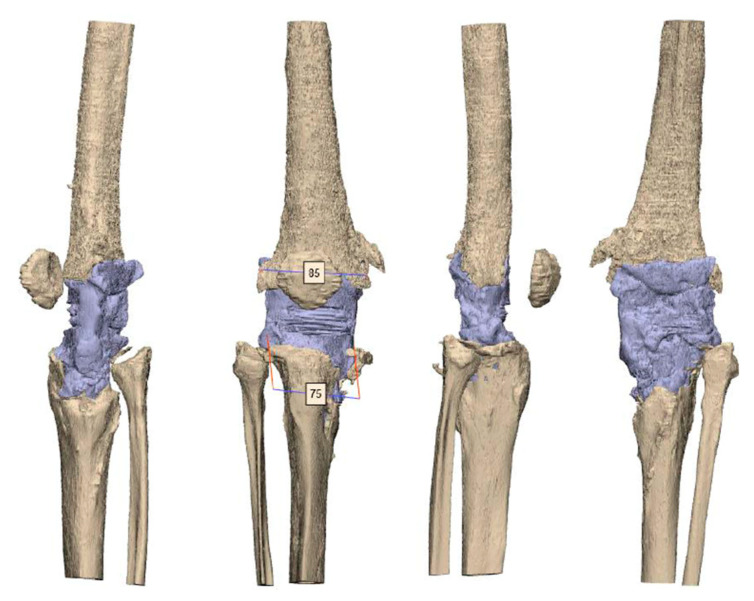
CT scan after the explantation of the septic prosthesis with a huge bone defect on the metaphysis of the tibia and femur. The tuberositas tibiae is still intact. However, the medial tibia plateau is loss. AO fixature bars are used for the rigid spacer. The mediolateral size for the femur is 85 mm and for the tibia 75 mm.

**Figure 3:**
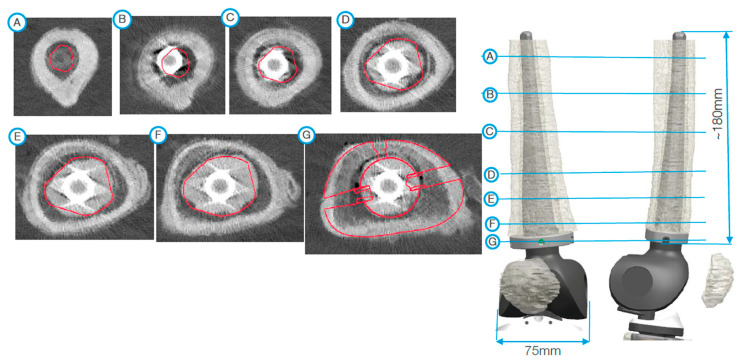
Planning of the femur component. For the diaphysis, an individual stem was planned. This is oval and takes up the natural curvature of the femur. Because of the defect of the metaphysis, a distal femoral replacement is used. The subfigures on the left side (A, B, C, …, G) demonstrate the axial view of the femur from the corresponding markings on the right side.

**Figure 4:**
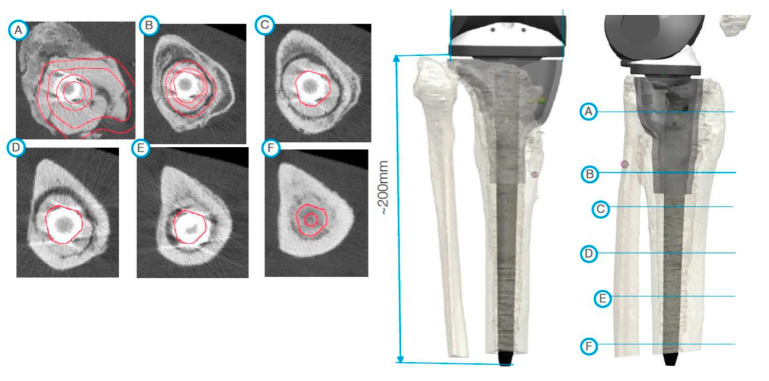
During planning, special consideration had to be given to the large defect of the medial tibial plateaus. The subfigures on the left side (A, B, C, …, F) demonstrate the axial view of the tibia from the corresponding markings on the right side.

**Figure 5:**
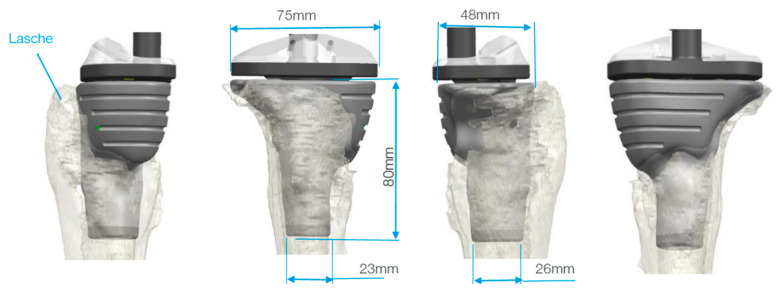
The customized cone has a porous structure in the area of bone contact and prefabricated grooves for possible osteosynthesis using cerclages.

**Figure 6:**
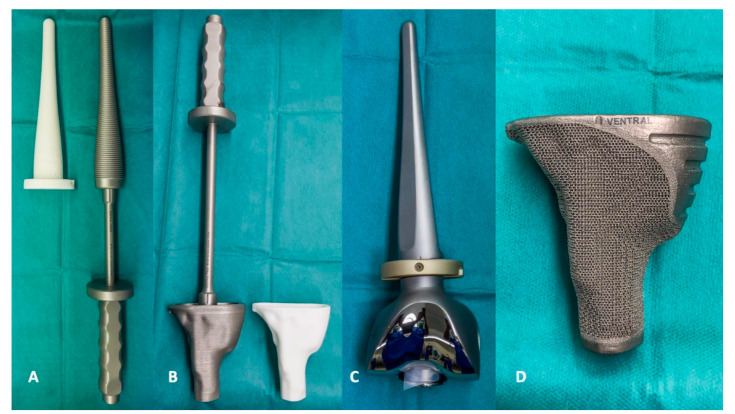
(**A**,**B**) A special rasp or impactor is supplied with each implant. The fit of the implant can be tested with a 3D dummy. (**C**) In planning, attention should be paid to the method of fixing the shafts. In this case, a cemented version was chosen. (**D**) Individual cones show improved biomechanics, as the force application into the bone is much more homogeneous. Each implant can be equipped with special features, such as fixation guides for osteosyntheses.

After segmentation, the first step of planning is the analysis of the bone defect (Figure 2). If the defect of the distal femur is too devastating, resection of the distal femur may be necessary. In such a scenario, the use of a conical oval stem is advisable (Figure 3). If the tibial tuberosity is still intact, proximal tibial replacement may not be required. With the help of a tibial cone, a standard tibial implant can be used. The cone provides a bearing surface to reconstruct the joint line (Figure 4). If iatrogenic fractures occurred during the first operation, this should be taken into account when planning the stem length. Features such as notches for cerclages or additional attachment options for the extensor mechanism are possible after consultation with the engineers (Figure 5 and Figure 6D).

**Figure 7:**
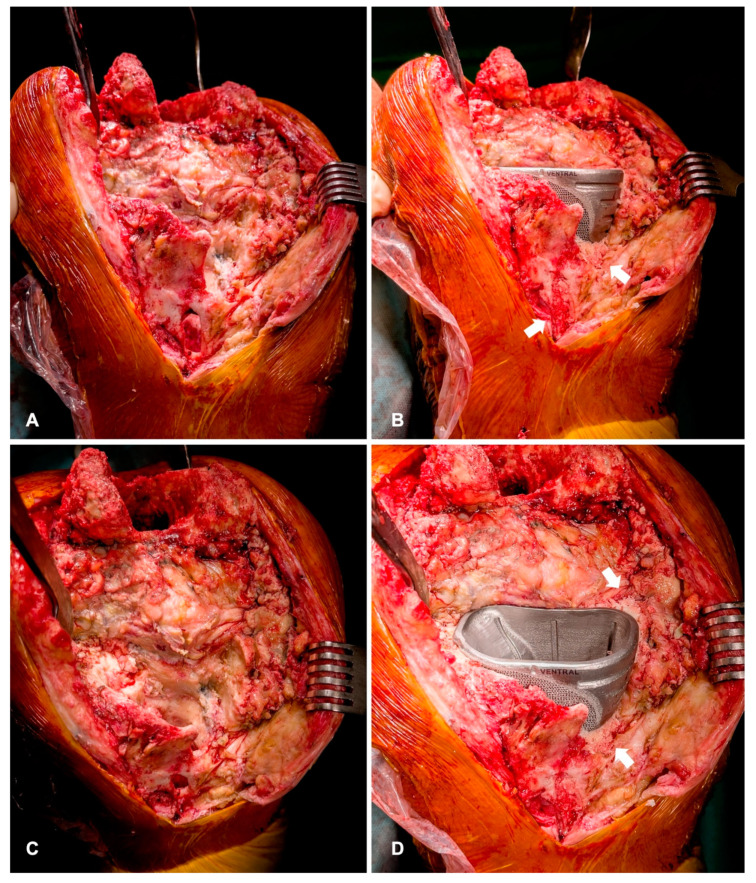
(**A**,**C**) Intraoperative findings of severe defect of the tibial metaphysis. The complete medial plateau is loss. (**B**,**D**) After cementless implantation of the cone, defects are filled with bone-impaction grafting (arrows). This leads to secondary osteointegration and partial reconstruction of the bone defect.

During the surgery, the bone bed is prepared with custom-made rasps, respectively, and impactors (Figure 6A,B). Trial implants can be used to check the position and progress of the preparation. The bone surface must be cleaned of any soft tissue such as pseudomembranes or cement residues to enable the best possible osteointegration to the cone surface. Bone-impaction grafting can be used to fill the remaining defects (Figure 7). The stems are usually cemented (Figure 1B).

## 3. Results

All demographics and indications are shown in Table 1. Six patients had a PJI and four an aseptic loosening (AL). The mean follow-up was 21 months (range: 12–40). The mean time between the two-stage surgeries was 71.6 days. No fractures were observed intra- and postoperatively. One patient was revised due to persistent hematoma (three weeks post-surgery) and one patient due to persistent PJI. This patient was treated with a change of polyethylene (three months post-surgery) and permanent antibiotic suppression therapy (cotrimoxazole). The type of implants, the postoperative range of motion, and the individual costs are listed in Table 2. Five patients received a distal femoral replacement (DFR) and no one needed a proximal tibial replacement. The mean preoperative VAS scale was 8.1 points (range: 7–9) and the mean postoperative VAS scale was 2 points (range: 0–5). The mean preoperative HKA was 177.2° varus (range: 176–181°) and the mean postoperative HKA was 179.2° varus (range: 178–180°). The mean passive postoperative range of motion was 92° (range: 80–110°). No extension deficit or extensor lag was observed.

## 4. Discussion

The most important finding of this study is that individual custom-made implants in rTKA are a safe procedure for limb salvage when standard implants are not suitable anymore. With the help of the individual tibial cones, the tuberosity tibiae with the extensor mechanism can be preserved. This leads to a good functional outcome and range of motion.

Megaprostheses have a high risk for early mechanical complications and infections followed by amputations [18]. We present an early revision rate of 20%. One patient was revised due to a persistent hematoma after three weeks and one due to a persistent PJI with the need for an exchange of the polyethylene and a permanent antibiotic suppression therapy. During the revision of this patient the same pathogen as in the previous surgeries was detected (multi-resistant *Staphylococcus capitis* and *Proteus mirabilis*). This is comparable to the current literature of outcome after implanting megaprostheses. Fraser et al. reported a revision-free survival of 58% after eight years in 247 cases with a rotating-hinge megaprosthesis [19]. Höll et al. reported a mid-term revision rate of 55% after a mean follow-up of 34 months (range: 10–84 months) [17]. Vertesich et al. reported a revision-free survival of distal femoral replacement of 74.8% at one year, 62.5% at three years, and 40.9% at ten years postoperatively [20]. Smith et al. demonstrated a complication rate at two years follow-up of 34% in a septic and aseptic mixed cohort [21]. Von Hintze et al. reported that PJI was the most common cause for revision after implanting rotating-hinge prostheses at a mid-term follow-up [22]. It is known that silver coating can reduce the revision rate after implanting megaprostheses in the case of PJI [23]. However, no anti-septic coatings were used in this study. The long-term results of our cohort concerning loosening rate remain to be seen. Evidence for superiority in terms of stability or survival of oval cemented stems over standard stems is not currently available. However, an advantage over standard implants is certainly possible.

The rate of intraoperative fractures regarding cone preparation and insertion for standard implants is very low. In a systematic review from 2018, Divano et al. observed an intraoperative fracture cones-related rate of 0.89% [13]. A recent systematic review from 2020 evaluated 927 cones and reported an intraoperative fracture rate of 1.2 ± 4.8% [24]. This suggests that those types of implants are safe to use in principle. Burastero et al. evaluated eleven patients with 16 custom-made cones regarding the clinical and biomechanical outcome. They reported no intraoperative fractures and no component migration after a mean follow-up of 26 months. Further, the authors demonstrated that custom-made cones induce a more homogeneously distributed bone stress compared to standard cemented or cementless stems in a finite element analysis [16]. We observed comparable results and had no fractures in this case series.

In severe metaphyseal tibial bone defects, proximal tibial replacement (PTR) has to be taken into account. One of the major issues of this treatment method is the comparatively poor function, especially a possible extensor lag. Fram et al. reported in a small case series an extensor lag in almost all patients [25]. Biau et al. reported a failure of the extensor mechanism in 26% of all patients with PTR after bone tumor resection [26]. However, the comparability between tumor resection and condition after failed knee replacement is not given. We could demonstrate a good postoperative function with a mean passive flexion of 92° and no extension deficit or extensor lag due to the preservation of the tuberosity tibiae and the natural extensor mechanism. There is good evidence for reducing the infection rate with the help of medial gastrocnemius muscle flap after PTR [27,28,29]. This primarily refers to PTR after malignant bone tumors, however, these results can be drawn with severe defects especially in the medial proximal tibia area after revision arthroplasty. In the present study, flap coverage was not necessary, but this should always be considered and evaluated individually.

A major point of criticism of custom-made implants is the prolonged time between the two procedures to plan and manufacture the implant. The mean interval between the removal and replacement was 71.6 days (range: 44–110 days). From our point of view, this correlates with the extent of bone loss, the number of implants to be fabricated, and the complexity of the case. In a case as described in 2.4. (Figure 1 and Figure 2), an intensive dialogue with the corresponding engineer is necessary. If only a tibial conus is needed, a six-week interval is possible (Table 2). Thus, the recommended six- to eight-week interval cannot always been adhered to [30]. However, due to the primary goal of joint preservation, this is accepted from the authors’ point of view. Nevertheless, the period from the removal procedure to the CT scan and production of the implant has the greatest potential for improvement in the future. Despite the extended interval, the results of this study are comparable to those in the literature. Winkler et al. reported a mean interval from explantation to implantation in a long-interval group of 63 days (range: 28–204 days) [31].

Another crucial point are the high implant costs. The mean implant cost in this study was EUR 10.739,92 per patient. However, the range is wide (EUR 4.321,53 to 19.900,82) and depends on the individual bone defect with the need for different implants such as cones or stems. However, these costs also include custom-made instruments such as raps or impactors (Figure 6A,B) that can only be used for the specific case.

There are several limitations to this study. This is a case series with a low volume of patients. Due to the very modern and young procedure of 3D printing with high costs, the extent of usage is limited. However, the caseload has been increasing in the past few years. Another reason for the low number of patients is the cost factor of implants. These are not fully reflected by the DRG of the national health insurance, which makes the use of such implants the last resort. Another point of criticism is the lack of a control group. However, due to the strict inclusion criteria with major bone defects, standard megaprostheses with a proximal tibial replacement have a severe functional disadvantage and randomization cannot be performed based on the ethical standards. Due to low volume of patients, we did not compare different types of implant coating. Especially in case of PJI, an antiseptic coating like silver could reduce the revision rate. Furthermore, we presented an early follow-up with no clinical data and no patient reported outcome parameters. However, the primary aim of this study was to investigate the early revision rate of these limb-salvage procedures. Further prospective studies are required to analyze the clinical outcomes and mid- and long term follow-up.

## 5. Conclusions

Individual custom-made implants for rTKA provide a safe procedure for patients with huge bone defects after several pre-surgeries. If standard knee systems with standard cones or sleeves are not suitable anymore, custom-made treatment offers the patient the last option for limb preservation. However, this is associated with increased costs.

## Figures and Tables

**Table 1:** Age, BMI, and the days of the interval between the two-stage procedures were listed as well as the number of pre-surgeries and pre-implants of each patient. Pre-surgeries comprise all procedures in the past including changes of metal components or soft tissue revisions. Pre-implants comprise all protheses including primary and revision TKA in the patient’s past. (ID: patient number, PJI: periprosthetic joint infection, AL: aseptic loosening).

ID	Age	BMI	Interval (Days)	Pre-Surgeries	Pre-Implants	Indication
1	69	25.7	67	3	2	PJI
2	61	26.5	78	7	3	PJI
3	70	26.8	73	7	4	AL
4	62	34.1	110	5	3	PJI
5	60	28.7	50	4	4	AL
6	79	21.6	57	5	3	AL
7	77	35.2	83	3	2	PJI
8	59	34.1	70	8	3	PJI
9	72	40.4	44	7	4	PJI
10	75	29.0	84	3	2	AL
Mean	68.4	30.2	71.6	5.2	3	

**Table 2:** The knee system, the amount and type of the specific individual implants, and the total costs are listed. (ID: patient number, DFR: distal femoral replacement, RTH: rotating hinge prosthesis, Ind: individual, Std: standard).

	Knee System		Femur Implants		Tibia Implants		Total Costs €
ID	Femur	Tibia	Ind. Cone	Ind. Stem	Ind. Cone	Std. Cone	
1	DFR	RTH	No	Yes	No	Yes	12.039,45
2	DFR	RTH	Yes	No	Yes	No	13.166,58
3	DFR	RTH	No	Yes	Yes	No	16.476,88
4	RTH	RTH	No	Yes	Yes	No	9.969,62
5	RTH	RTH	No	No	Yes	No	5.131,78
6	DFR	RTH	No	No	Yes	No	8.772,03
7	RTH	RTH	Yes	No	Yes	No	11.842,35
8	DFR	RTH	No	Yes	Yes	No	19.900,82
9	RTH	RTH	No	No	Yes	No	4.321,53
10	RTH	RTH	No	No	Yes	No	5.778,14
Mean							10.739,92

## Data Availability

The data presented in this study are available in this article. Further datasets of this study are available from the corresponding author on reasonable request.
